# Potential Association between the Use of Anabolic Steroids and COVID-19 Infection

**DOI:** 10.3390/healthcare10020196

**Published:** 2022-01-20

**Authors:** Yusuf S. Althobaiti, Mohammed S. Alzahrani, Shahad M. Alhumayani, Shaima A. Assiry, Hadeel F. Aljuaid, Majed A. Algarni

**Affiliations:** 1 Department of Pharmacology and Toxicology, College of Pharmacy, Taif University, P.O. Box 11099, Taif 21944, Saudi Arabia; salhumayani@gmail.com (S.M.A.); pharmaspring111@gmail.com (S.A.A.); Hadeel.f.Aljuaid@gmail.com (H.F.A.); 2 Addiction and Neuroscience Research Unit, Taif University, P.O. Box 11099, Taif 21944, Saudi Arabia; 3 Department of Clinical Pharmacy, College of Pharmacy, Taif University, P.O. Box 11099, Taif 21944, Saudi Arabia; M.s.alzahrani@tu.edu.sa (M.S.A.); m.alqarni@tu.edu.sa (M.A.A.)

**Keywords:** anabolic steroids, androgenic, COVID-19, coronavirus, athletes

## Abstract

Anabolic androgenic steroids (AASs) are synthetic analogs of testosterone that can affect the immune system. Bodybuilders and sportsmen are at risk of abusing AASs. The aim of this study was to investigate the association between AASs use and coronavirus disease (COVID-19). This cross-sectional study included adults aged 18 years and above. Between 16 April and 23 June 2021, gym-attending participants completed an online survey. Multivariable analysis was performed using multiple logistic regression to identify factors associated with COVID-19 diagnosis and severity. Current use of AASs was reported in 7.5% of the 520 study participants. Approximately 20% of the study participants reported that they had contracted COVID-19, approximately half of whom reported moderate to severe disease. Contracting COVID-19 was reported more frequently by current users than by non-current users (35.90% vs. 18.92%, *p* = 0.011). Multivariable analysis revealed that contracting COVID-19 was nearly five times more likely among current users of AASs than among non-current users (OR = 4.89, 95% CI: 1.69–14.13). Current use of AASs was also associated with greater odds of moderate to severe COVID-19 disease (OR = 3.71, 95% CI: 1.04–13.21). Our findings suggest that the use of AASs could be an underlying risk factor for COVID-19 severity.

## 1. Introduction

Anabolic androgenic steroid (AAS) drugs are composed of synthetic testosterone (male sex hormone) derivatives that have both anabolic effects combined with androgenic effects by binding to the active site on androgen receptors. AAS drugs are usually prescribed by physicians for a list of indications, including cases of certain types of anemia, androgen insensitivity syndromes, some forms of breast cancer, angioedema and weight gain after serious illness [1,2]. There are no recorded cases of abuse or dependence in patients using therapeutic doses of AASs for legitimate indications. However, AASs are widely abused by bodybuilders and athletes and are not prescribed to healthy people [3]. AASs are abused by athletes who aim to improve their physical appearance and performance; however, it misuse is accompanied by several physical and psychiatric side effects, including gynecomastia and baldness in male users [4], higher risk of liver neoplasms and heart disease [5], depression, mania, psychosis and aggression [6,7,8,9,10]. Moreover, AAS use has been shown to affect the immune system resulting, in either immunostimulatory or immunoinhibitory effects, depending on the steroid nucleus; AASs with alterations to the steroid nucleus stimulate the proliferation of T cells as well as other immune cells [11,12]. In contrast, AASs with a preserved steroid nucleus have immunosuppressive effects as they reduce the number of immune cells and their functions [12]. These effects are more obvious following exposure to higher doses of these drugs [11,12].

Of note, by the end of 2019, an outbreak of acute respiratory syndrome of unknown origin was detected in Wuhan, Hubei Province, China [13]. Globally, the prevalence of COVID-19, its rapid transmission and increasing death rates due to the disease led the World Health Organization to announce a pandemic on 12 March 2020 [13]. In the flowing months after identification of the initial cases, COVID-19 spread to 171 countries and there were around 215,546 confirmed cases by 19 March 2020 [14]. Many COVID-19 patients were asymptomatic; however, the most common symptoms experienced by 50% of COVID-19 patients were fever, sore throat, cough, fatigue, headache and myalgia or arthralgia [15].

COVID-19 infection can affect the immune system causing lymphopenia, which is defined as a decrease in the number of blood lymphocytes, implying that cellular immune responses have been suppressed. Human immune responses to SARS-CoV and MERS-CoV have been carefully researched; however, studies on SARS-CoV-2 have yet to be thoroughly explored. The virus induces a plethora of cytokines in the most severe cases of COVID-19, which are identified by activated T-helper-1 (Th1) cell responses with elevated levels of interleukins-1b, 2, 6, 7, 8 and 10 (IL-1b, IL-2, IL-6, IL-7, IL-8 and IL-10), as well as tumor necrosis factor-a (TNF-a), granulocyte-colony stimulating factor (GSF), interferon-g, induced protein (IP-10), monocyte chemoattractant protein-1 (MCP-1) and macrophage inflammatory-protein 1-a (MIP-1a). Increased amounts of proinflammatory cytokines can cause pulmonary inflammation and tissue damage in the heart, liver and kidneys, which can lead to multiorgan failure. In patients with SARS-CoV, MERS-CoV, or SARS-CoV-2 infection, an excess of cytokines has been linked to the onset and progression of ARDS. A considerable increase in death rate is also linked to a dysregulated cytokine response in severe COVID-19 cases with ARDS, especially in older patients [16].

In Saudi Arabia, the prevalence of AAS use has been investigated by cross-sectional studies in the capital city of Riyadh in 2015 and Jazan city in 2017; these studies included 600 athletes in Riyadh and 465 bodybuilders in Jazan City. The studies showed that 30.5% of the study participants in Riyadh [17] and 38.7% of those in Jazan [18] were AAS abusers. Since there is a high prevalence of AAS use in the Kingdom of Saudi Arabia (KSA), recent studies have shown an association of AAS use with coronavirus disease (COVID-19) [17,18,19]. The hypothesis is that the illegal use of AASs by bodybuilding may worsen COVID-19 symptoms; this may be an important issue affecting young adults and athletes worldwide since the prevalence of illegal AAS use is increasing. This study aimed to observe COVID-19 progression in AAS users infected with severe acute respiratory syndrome coronavirus 2 (SARS-CoV-2) in Saudi Arabia.

## 2. Materials and Methods

### 2.1. Study Design and Setting

This cross-sectional study was conducted between 16 April and 23 June 2021. The target study population included men and women aged 18 years and older who attended gyms in the Makkah region. During the study period, a link to the online survey was promoted at local gyms and fitness centers and shared via social media. The online survey was designed with an option that ensured that each respondent could participate only once. All respondents were informed about the study’s purpose and were assured of the anonymity of their responses. This study was approved by the Scientific Research Ethics Committee of Taif University.

### 2.2. Survey Tool

In this study, we used a standardized self-administered survey tool. The survey included items about demographics, in-person work or school (never, sometimes, always), the use of AASs (current use, past use, non-use) and the presence of any chronic diseases. In addition, the survey involved items relating to whether the participants had contracted COVID-19, whether a family member had contracted COVID-19, severity of the disease and the symptoms encountered during the illness. The severity of COVID-19 was self-reported and categorized into asymptomatic, mild (home care), moderate (outpatient care) and severe (inpatient care), based on previously published guidelines [20,21]. For the purpose of this study, severity was collapsed into two categories: asymptomatic/mild and moderate/severe. The survey was evaluated for face validity by two faculty members who had knowledge of the subject. The survey was then pilot-tested on a small number of subjects to clarify the items.

### 2.3. Statistical Analysis

Univariate, bivariate and multivariate analyses were performed. Frequencies and percentages were calculated for categorical variables and means with standard deviations were calculated for numerical variables. Bivariable associations with COVID-19 diagnosis and severity were assessed using Pearson’s Chi-squared test or Fisher’s exact test, as appropriate. Variables that were significantly associated with outcomes (*p* < 0.05) were further evaluated in the adjusted models. Two multiple logistic regression models were built for each outcome: COVID-19 diagnosis and disease severity. Odds ratios [OR], 95% confidence intervals (CIs) and *p*-values were calculated and used to determine the significance of associations. All analyses were performed using SAS University Edition (SAS Institute, Cary, NC, USA).

## 3. Results

Overall, 520 participants completed the survey. The characteristics of the participants are listed in Table 1. Of the 520 participants, 294 (56.54%) were female, the mean age was 27.49 ± 9.09 years, 39 (7.50%) were current users of AASs and 68 (13.08%) were previous users. Approximately 20% of participants (*n* = 105) reported contracting COVID-19, with almost half reporting moderate to severe disease.

In comparison to female participants, male participants had a higher prevalence of COVID-19 (28.76% vs. 13.61%, *p* ≤ 0.001) and higher prevalence of moderate to severe disease (14.16% vs. 7.14%, *p* = 0.009) (Table 2). Current users of AASs had a higher prevalence of COVID-19 (35.90% vs. 18.92%, *p* = 0.011) and higher disease severity (17.95% vs. 9.56%, *p* = 0.101) compared non-current users. Participants who reported that a family member had COVID-19 had a higher prevalence of COVID-19 (39.66% vs. 4.51%, *p* < 0.001) and higher disease severity (21.12% vs. 1.39%, *p* < 0.001). Participants with comorbidities had a higher prevalence of moderate to severe COVID-19 (19.15% vs. 9.30%, *p* = 0.042) than those with no comorbidities.

**Figure 1:**
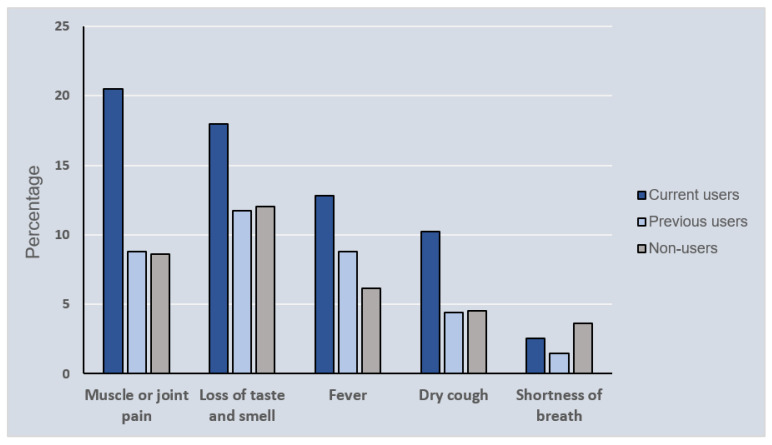
Most frequently self-reported COVID-19 symptoms, stratified by anabolic steroids use.

Figure 1 shows the most common self-reported COVID-19 symptoms stratified by the use of AASs. All COVID-19 symptoms, with the exception of shortness of breath, were reported more frequently by current users than past or never users. The most prevalent symptom described by current users was muscular or joint discomfort, accounting for approximately 21% of the current users. In comparison, less than 10% of past users and non-users reported muscular or joint discomfort.

Table 3 shows the results of the multiple logistic regression analysis of contracting COVID-19. Participants aged 18–24 years had greater odds of contracting COVID-19 compared to those over the age of 35 years (adjusted OR = 2.33, 95% Cl: 1.22–4.44). Male participants were more likely to contract COVID-19 than females (adjusted OR = 3.34, 95% CI: 1.83–6.07). The multivariable analysis revealed that being a current user of AASs was associated with greater odds of contracting COVID-19 (adjusted OR = 4.89, 95% CI: 1.69–14.13), while past AAS use was not associated with contracting COVID-19 (adjusted OR = 0.59, 95% CI: 0.24–1.43). The adjusted model also showed that having a family member who was infected with COVID-19 was associated with greater odds of contracting COVID-19.

Multiple logistic regression analysis of moderate to severe COVID-19 is shown in Table 4. Current AAS users were more likely to report moderate to severe symptoms of COVID-19 than non-users (adjusted OR = 3.71, 95% CI: 1.04–13.21). The adjusted model also showed that males, those who reported that a family member had contracted COVID-19 and participants with comorbidities had greater odds of having moderate to severe symptoms of COVID-19.

## 4. Discussion

This study aimed to observe COVID-19 progression in AAS users infected with severe acute respiratory syndrome coronavirus 2 (SARS-CoV-2) in Saudi Arabia. Based on the findings of this study, the data can be interpreted as follows: consumption of AASs may affect COVID-19 risk, symptom severity and the nature of the disease. Male participants had a higher risk of COVID-19 and higher severity of symptoms compared to female participants. This matches trends in the general population, where males are at a greater risk of contracting COVID-19. The most commonly reported COVID-19 symptom among AAS users was muscle or joint pain. The most common symptom reported by nonusers and previous users was loss of taste and smell. The severity of symptoms in participants with COVID-19, who were current users of AASs, was almost two times worse than that in previous users or non-users. These results may be a consequence of the effects of AASs on the immune system [11,12], which is directly responsible for the presentation and severity of symptoms of COVID-19 [22]. In addition, the genetic differences between the immune systems in men and women may lead to more susceptibility of men to severe acute infection compared to women and these differences can be seen in boys and girls even before puberty [23].

The first and only case report that linked COVID-19 symptom severity and AAS use was reported by Cadegiani et al., in which a healthy 28-year-old man presented at a hospital with severe COVID-19 symptoms [24]. Upon investigation, researchers found that the patient had been taking oxandrolone 40 mg/day to enhance his body building for the past 30 days [24].

Since AAS can affect the immune system, some studies have investigated the association between AAS use and COVID-19 [19]. In Yale New Haven Hospital and UK Biobank, an association between androgen imbalance and complications of COVID-19 have been identified [19]. Inhibitors of 5 alpha reductases that inhibit androgen signaling can reduce ACE2 levels and thus decrease viral spike- receptor-binding domain internalization [19]. In another study, it was suggested that COVID-19 risks could be reduced in the elderly by hormone replacement therapy (estrogen and testosterone) due to the anti-inflammatory actions of sex hormones and the anabolic actions of testosterone [22]. Another study showed that the COVID-19 pandemic had clearly affected the use of AAS by reducing AAS doses and training frequency as compared to pre-pandemic [25].

Our study had several limitations. First, the prevalence of the targeted study group (AAS current users with COVID-19) was limited (*n* = 14); a larger prevalence would give more accurate interpretations. Second, this was a cross-sectional study and data were collected only once, with no follow-up to assess future developments in the participants, which may have revealed additional knowledge. Additionally, because we lacked longitudinal data, causality could not be assessed. Therefore, it is recommended that a prospective cohort study be conducted in the future. Third, as with any self-reported data, there is always the potential risk of recall bias and response bias. Finally, this study was conducted within a period during which vaccines were not widely available and we did not collect data on whether respondents were vaccinated. In spite of these limitations, this study is among the first to explore the association between AAS use and COVID-19 infection.

## 5. Conclusions

This research examined the effects of AAS use on COVID-19 infection. It is a topic that has not been researched sufficiently and more research and controlled trials should be conducted to investigate the pathophysiological correlation between the virus and AAS drugs, including how the pharmacodynamics and pharmacokinetics of AASs are altered during a COVID-19 infection. This study suggests that the use of AAS could be an underlying risk factor for COVID-19 severity.

## Figures and Tables

**Table 1:** Participants’ Characteristics.

Characteristic	*n* = 520
Gender, *n* (%)	
Female	294 (56.54)
Age, mean (SD)	27.49 (9.09)
Age groups, *n* (%)	
18–24 years	235 (45.19)
25–34 years	138 (26.54)
≥35 years	147 (28.27)
In-person work/school, *n* (%)	
Never	147 (28.27)
Sometimes	163 (31.35)
Always	210 (40.38)
Use of anabolic steroids, *n* (%)	
Current use	39 (7.50)
Previous use	68 (13.08)
Never been used	413 (79.42)
A family member contracted COVID-19 (Yes), *n* (%)	232 (44.62)
Presence of comorbidity (Yes), *n* (%)	47 (9.04)
Contracted COVID-19 (Yes), *n* (%)	105 (20.19)
Self-reported moderate to severe COVID-19 (Yes), *n* (%)	53 (10.19)

COVID-19, coronavirus disease; SD, standard deviation.

**Table 2:** Bivariable analysis of contracting COVID-19 and the severity of the disease.

Characteristic	Contracted COVID-19 (Yes), %	*p* *	Moderate\Severe COVID-19 (Yes), %	*p* *
Gender		<0.001		0.009
Male	28.76		14.16	
Female	13.61		7.14	
Age group		0.538		0.601
18–24 years	22.13		11.06	
25–34 years	17.39		7.97	
≥35 years	19.73		10.88	
Past use of anabolic steroids		0.289		0.373
No	19.47		9.73	
Yes	25.00		13.24	
Current use of anabolic steroids		0.011		0.101
No	18.92		9.56	
Yes	35.90		17.95	
A family member contracted COVID-19		<0.001		<0.001
No	4.51		1.39	
Yes	39.66		21.12	
Presence of comorbidity		0.565		0.042
No	19.87		9.30	
Yes	23.40		19.15	
In-person work or school		0.051		0.052
Never	14.29		5.44	
Sometimes	19.63		10.43	
Always	24.76		13.33	

COVID-19, coronavirus disease * *p*-values produced using Pearson’s chi-squared or Fisher’s exact test.

**Table 3:** Multiple logistic regression analysis of contracting COVID-19.

Factors	Unadjusted OR (95% CI)	*p*	Adjusted OR (95% CI)	*p*
Gender				
Female	Ref		Ref	
Male	2.56 (1.65–3.98)	<0.001	3.34 (1.83–6.07)	<0.001
Age group				
≥35 years	Ref		Ref	
25–34 years	0.86 (0.47–1.55)	0.310	0.99 (0.49–2.00)	0.173
18–24 years	1.16 (0.69–1.92)	0.381	2.33 (1.22–4.44)	0.004
Past use of anabolic steroids				
No	Ref		Ref	
Yes	1.38 (0.76–2.50)	0.290	0.59 (0.24–1.43)	0.246
Current use of anabolic steroids				
No	Ref		Ref	
Yes	2.40 (1.20–4.79)	0.013	4.89 (1.69–14.13)	0.003
A family member contracted COVID-19				
No	Ref		Ref	
Yes	13.90 (7.51–25.71)	<0.001	16.93 (8.72–32.85)	<0.001
Presence of comorbidity				
No	Ref		Ref	
Yes	1.23 (0.61–2.51)	0.566	1.32 (0.58–3.00)	0.506
In-person work or school				
Never	Ref		Ref	
Sometimes	1.47 (0.80–2.67)	0.862	1.37 (0.66–2.84)	0.663
Always	1.98 (1.13–3.45)	0.027	1.46 (0.71–2.99)	0.427

COVID-19, coronavirus disease; OR = odds ratio; CI = confidence interval; Ref = reference.

**Table 4:** Multiple logistic regression analysis of moderate to severe COVID-19.

Factors	Unadjusted OR (95% CI)	*p*	Adjusted OR (95% CI)	*p*
Gender				
Female	Ref		Ref	
Male	2.14 (1.20–3.83)	0.01	2.13 (1.03–4.38)	0.04
Age group				
≥35 years	Ref		Ref	
25–34 years	0.71 (0.32–1.58)	0.322	0.85 (0.35–2.06)	0.259
18–24 years	1.02 (0.53–1.97)	0.515	1.79 (0.82–3.89)	0.063
Past use of anabolic steroids				
No	Ref		Ref	
Yes	1.41 (0.66–3.04)	0.376	0.66 (0.22–2.01)	0.463
Current use of anabolic steroids				
No	Ref		Ref	
Yes	2.07 (0.87–4.95)	0.103	3.71 (1.04–13.21)	0.043
A family member contracted COVID-19				
No	Ref		Ref	
Yes	19.01 (6.75–53.57)	<0.001	20.93 (7.23–60.57)	<0.001
Presence of comorbidity				
No	Ref		Ref	
Yes	2.31 (1.05–5.08)	0.038	2.69 (1.10–6.58)	0.03
In-person work or school				
Never	Ref		Ref	
Sometimes	2.02 (0.85–4.83)	0.518	2.03 (0.77–5.38)	0.415
Always	2.67 (1.18–6.04)	0.036	2.28 (0.88–5.86)	0.185

COVID-19, coronavirus disease; OR = odds ratio; CI = confidence interval; Ref = reference.

## Data Availability

Data are available upon reasonable request.

## References

[1] Conway A.J., Handelsman D.J., Lording D.W., Stuckey B., Zajac J.D. (2000). Use, Misuse and Abuse of Androgens. The Endocrine Society of Australiaconsensus Guidelines for Androgen Prescribing. Med. J. Aust..

[2] Shahidi N.T. (2001). A Review of the Chemistry, Biological Action, and Clinical Applications of Anabolic-Androgenic Steroids. Clin. Ther..

[3] Anabolic Steroids: Uses, Abuse, and Side Effects. WebMD. https://www.webmd.com/men/anabolic-steroids.

[4] Torres-Calleja J., González-Unzaga M., DeCelis-Carrillo R., Calzada-Sánchez L., Pedrón N. (2001). Effect of Androgenic Anabolic Steroids on Sperm Quality and Serum Hormone Levels in Adult Male Bodybuilders. Life Sci..

[5] Zaugg M., Jamali N.Z., Lucchinetti E., Xu W., Alam M., Shafiq S.A., Siddiqui M.A. (2001). Anabolic-Androgenic Steroids Induce Apoptotic Cell Death in Adult Rat Ventricular Myocytes. J. Cell. Physiol..

[6] Thiblin I., Lindquist O., Rajs J. (2000). Cause and Manner of Death Among Users of Anabolic Androgenic Steroids. J. Forensic Sci..

[7] Pope H.G., Brower K.J., Sadock B.J., Sadock V.A. (2000). Anabolic-Androgenic Steroid Abuse. Comprehensive Textbook of Psychiatry.

[8] Bahrke M.S., Yesalis C.E. (2000). Psychological Effects of Endogenous Testosterone and Anabolic-Androgenic Steroids. Anabolic Steroids in Sport and Exercise.

[9] Pope H.G., Kouri E.M., Hudson J.I. (2000). Effects of Supraphysiologic Doses of Testosterone on Mood and Aggression in Normal Men: A Randomized Controlled Trial. Arch. Gen. Psychiatry.

[10] Midgley S.J., Heather N., Davies J.B. (2001). Levels of Aggression Among a Group of Anabolic-Androgenic Steroid Users. Med. Sci. Law.

[11] Wyle F.A. Immunosuppression by Sex Steroid Hormones. The Effect Upon PHA- and PPD-Stimulated Lymphocytes. https://www.ncbi.nlm.nih.gov/pmc/articles/PMC1540928/.

[12] Marshall-Gradisnik S., Green R., Brenu E., Weatherby R. (2009). Anabolic androgenic steroids effects on the immune system: A review. J. Open Life Sci..

[13] Ciotti M., Ciccozzi M., Terrinoni A., Jiang W.C., Wang C.B., Bernardini S. (2020). The COVID-19 pandemic. Crit. Rev. Clin. Lab. Sci..

[14] Dietz L., Horve P.F., Coil D.A., Fretz M., Eisen J.A., Van Den Wymelenberg K. (2019). Novel Coronavirus (COVID-19) pandemic: Built environment considerations to reduce transmission. mSystems.

[15] Struyf T., Deeks J.J., Dinnes J., Takwoingi Y., Davenport C., Leeflang M.M., Spijker R., Hooft L., Emperador D., Dittrich S. (2020). Cochrane COVID-19 Diagnostic Test Accuracy Group. Signs and symptoms to determine if a patient presenting in primary care or hospital outpatient settings has COVID-19 disease. Cochrane Database Syst. Rev..

[16] Muralidar S., Ambi S.V., Sekaran S., Krishnan U.M. (2020). The emergence of COVID-19 as a global pandemic: Understanding the epidemiology, immune response and potential therapeutic targets of SARS-CoV-2. Biochimie.

[17] Jabari M., Al-shehri H., Al-faris A., Al-sayed M., Algaeed F., Al-sobaie N., Al-saleh F. (2016). The Prevalence of Anabolic Androgenic Steroid Use Amongst Athletes in Riyadh (Saudi Arabia). Electron. Physician.

[18] Bahri A., Mahfouz M.S., Marran N.M., Dighriri Y.H., Alessa H.S., Khwaji M.O., Zafar S.M. (2017). Prevalence and Awareness of Anabolic Androgenic Steroid Use Among Male Body Builders in Jazan, Saudi Arabia. Trop. J. Pharm. Res..

[19] Strope J.D., Chau C.H., Figg W.D. (2020). Are Sex Discordant Outcomes in COVID-19 Related to Sex Hormones?. Semin. Oncol..

[20] Varghese G.M., John R., Manesh A., Karthik R., Abraham O.C. (2020). Clinical management of COVID-19. Indian J. Med. Res..

[21] Son K.B., Lee T.J., Hwang S.S. (2021). Disease severity classification and COVID-19 outcomes, Republic of Korea. Bull. World Health Organ..

[22] Brodin P. (2021). Immune Determinants of COVID-19 Disease Presentation and Severity. Nat. Med..

[23] Webb K., Peckham H., Radziszewska A., Menon M., Oliveri P., Simpson F., Deakin C.T., Lee S., Ciurtin C., Butler G. (2019). Sex and Pubertal Differences in the Type 1 Interferon Pathway Associate With Both X Chromosome Number and Serum Sex Hormone Concentration. Front. Immunol..

[24] Cadegiani F., Lin E.M., Goren A., Wambier C.G. (2021). Potential Risk for Developing Severe COVID-19 Disease Among Anabolic Steroid Users. BMJ Case Rep..

[25] Zoob Carter B.N., Boardley I.D., van de Ven K. (2021). The Impact of the COVID-19 Pandemic on Male Strength Athletes Who Use Non-prescribed Anabolic-Androgenic Steroids. Front. Psychiatry.

